# Decolorization of Corn Fiber Arabinoxylan Extract with (MN102) Resin: Adsorption Performance and Film-Forming Capacity

**DOI:** 10.3390/polym17152128

**Published:** 2025-08-01

**Authors:** Verónica Weng, Diana Gago, Carla Brazinha, Vítor D. Alves, Isabel M. Coelhoso

**Affiliations:** 1 LAQV-REQUIMTE, Department of Chemistry, NOVA School of Science and Technology, NOVA University of Lisbon, 2829-516 Caparica, Portugal; v.weng@campus.fct.unl.pt (V.W.); dx.gago@campus.fct.unl.pt (D.G.); c.brazinha@fct.unl.pt (C.B.); 2 LEAF—Linking Landscape, Environment, Agriculture and Food, Instituto Superior de Agronomia, Universidade de Lisboa, Tapada da Ajuda, 1349-017 Lisbon, Portugal; vitoralves@isa.ulisboa.pt

**Keywords:** adsorption, arabinoxylan, arabinoxylan film, corn fiber, decolorization, macroporous resin

## Abstract

Arabinoxylan is a polysaccharide with film-forming properties, present in corn fiber, and a low-value by-product. The extract has a deep brown color, producing films of the same shade, which may not be appealing. This study addresses, for the first time, the adsorption of colored compounds present in an arabinoxylan extract using resin MN102. The resin successfully adsorbed the colored compounds from the arabinoxylan extract. After four consecutive adsorption/desorption cycles, the efficiency of the resin was similar, only decreasing from 63.3% to 52.9%. Langmuir and Freundlich models were fitted to the results of adsorption isotherm experiments, with the Freundlich model demonstrating the best fit to the experimental results. A fixed-bed column loaded with the resin was used for the removal of the colored compounds from the arabinoxylan extract, and the effect of the volumetric flow rate was investigated. The Yan and log-Gompertz models showed the best fit to the experimental breakthrough curves. This study systematically evaluated the adsorption conditions, providing a comprehensive analysis of the performance of the resin in the removal of the colored compounds. Additionally, the ability of the extract to maintain its film-forming properties after decolorization was evaluated, and some of the film’s key characteristics were evaluated, namely its color, solubility in water and mechanical properties.

## 1. Introduction

Hemicellulose, an abundant organic resource found in nature, consists of a group of polysaccharides that provide structure to cell walls. The main backbone can be substituted with xylose, glucose, mannose and galactose and branched with arabinose, galactose, glucose and glucuronic acid substituents, making their structure very diverse [1,2]. Arabinoxylan is a type of hemicellulose, composed of a β-(1,4)-D-xylopyranosyl main backbone, which can be substituted, and α-L-arabinofuranose units as the side groups. These side groups can also be found esterified to different hydroxycinnamic acid derivatives, especially to ferulic acid, a phenolic compound and precursor for the production of bio-vanillin [3,4,5]. Arabinoxylan has prebiotic and film-forming properties, and it is commonly found in various cereal grains in significant abundance [3,6,7].

Corn fiber is a by-product obtained from the wet milling of corn, mostly produced in the starch industry. It contains a fraction consisting mainly of the outer layers of the grain, enriched in arabinoxylan, and it is usually used as animal feed [3,6,8].

Under a circular economy approach, there are several methods for obtaining arabinoxylan-rich products, one of them being alkaline extraction. This type of extraction can efficiently recover arabinoxylan from various grains, including corn fiber, even under mild conditions. Besides arabinoxylan, this type of extract has a high content of phenolic compounds, namely ferulic acid, including its dimers, trimers and tetramers, and *ρ*-coumaric acid [3]. This blend of different and complex molecules may be responsible for the deep brown color of the extract [3].

When arabinoxylan extracts are used in the production of biodegradable films, their color is also deep brown, which may not be appealing for food packaging applications. Previous attempts to improve the color and transparency of arabinoxylan films have been described in the literature. Weng et al., 2021, reported oxidative decolorization of arabinoxylan with hydrogen peroxide, yielding light yellow films [6]. Egüés et al., 2014, also reported a similar arabinoxylan bleaching process, resulting in lighter films [9]. However, oxidant agents can react with polysaccharides and impact their physicochemical properties. For this reason, alternative efficient and environmentally friendly decolorization methods should be explored.

Adsorption is commonly used to recover solutes from diluted solutions, as well as to separate and enrich the desired components [9]. It has applications in the food and pharmaceutical sectors, wastewater treatment and chromatographic analysis [9].

Adsorption with resins has also been reported as a widely used method for the decolorization of polysaccharides [10]. Adsorption processes using macroporous resins are simple, easy to implement and to scale up and associated with a low initial cost. Resins are described as polymer adsorbents, having a porous network structure and a high surface area. This specific area is often bonded to other components, like polymeric monomers, crosslinking agents and pore-causing agents. For adsorption to occur, target compounds usually bond with the surface through physical and/or chemical interactions [10]. Additionally, resins can be recovered and used again in new adsorption processes [11,12].

Dong et al., 2021, decolorized red grape pomace polysaccharides employing the resin HPD-300 in a dosage range of 0.12–0.28 g mL^−1^, achieving a decolorization degree close to 37% for the lowest dosage (0.12 g mL^−1^) and around 63% for the optimized dosage (0.20 g mL^−1^) [13]. Wang et al., 2024 were able to extract polyphenols from *Inonotus hispidus* with a macroporous resin (MAR HPD-600) with 91% adsorption and 85% desorption degrees [14]. Li et al., 2024 enriched four diterpenoids from a crude extract of *Wikstroemia chamaedaphne* buds with a nonpolar D101 macroporous resin with an adsorption capacity of around 20 mg g^−1^ [15].

Valério et al., 2022, purified and recovered ferulic acid extracted from corn fiber by alkaline extraction with the resins PAD900, XAD 16N, XAD 7HP and MN102, obtaining the best results (around 90% recovery) with MN102 [11]. Their feed solution was obtained through a membrane purification process, with the permeate collected to produce biotechnological vanillin from the recovered ferulic acid.

Macronet™ MN102 is a commercially available macroporous resin with a tertiary amine group, which is technically functionalized with a low ion exchange capacity (0.15 eq L^−1^). This resin is reportedly used for the decolorization of sweeteners, beer broths and sugar solutions [16] and for the efficient removal and recovery of phenolic compounds from agave bagasse hydrolysates [17], as well as for the removal of limonin and polyphenols [18].

The present work shows the application of the resin MN102 in the process of arabinoxylan extract decolorization for the efficient removal of colored compounds. The resin was applied to a complex extract rich in ferulic acid and derivatives, as well as other colored compounds, previously obtained from corn fiber through alkaline extraction and further purified through membrane filtration [7]. The effect of the adsorption conditions, including the contact time, adsorbent dosage and initial solution concentration, was evaluated. Regeneration studies were conducted to determine the possibility of reutilization of the resin MN102 in the decolorization process. The adsorption efficiency was assessed within consecutive adsorption/desorption cycles, using ethanol as a desorbing agent. The experimental data were analyzed using equilibrium isotherms. Dynamic adsorption studies were conducted to evaluate the influence of the flow rate on the breakthrough curves, and various dynamic adsorption models were applied to interpret the experimental results and predict the relevant parameters. A scaled-up version of the process was also assessed. Lastly, the arabinoxylan recovered after the adsorption process was used to obtain films, which were characterized in terms of their color, water solubility and mechanical properties.

This study is the first to explore the application of the resin MN102 in the decolorization of arabinoxylan extracts. By systematically evaluating the adsorption conditions, the work provides a comprehensive analysis of the resin’s performance and reusability. The findings offer valuable insights concerning efficient strategies for the removal of colored compounds from agricultural by-products.

## 2. Materials and Methods

### 2.1. Materials

Resin MN102 was kindly provided by Purolite™ (Ecolab, Brașov, Romania); it is a hyper-crosslinked polystyrenic macroporous adsorbent resin with weak base functionality in its free base form (tertiary amine). It appears as small, brown spherical beads and is stable in a pH range of 0–14 and up to 60 °C. Pure ethanol (≥99.8%, Honeywell, Lisbon, Portugal) was used in the desorption process. Glycerol (≥99.0%, Honeywell) was used as a plasticizer. The arabinoxylan extract was obtained through a previously reported process [7]. Briefly, it was extracted from corn fiber with an alkaline solution and further purified through membrane processes, at the end of which the retentate was collected and freeze-dried [7].

### 2.2. Methods

#### 2.2.1. Resin Preparation

Resin MN102 was prepared as described by Valério et al., 2022 [11]. The resin was submerged in an ethanol–water mixture (1:1, *v*/*v*) with gentle stirring for 24 h to remove the preservatives. After this time, the excess liquid was decanted, and the resin was vacuum-filtered and washed with deionized water. The filtered resin was then stored in a plastic bottle until further use. The dry weight of the resin was determined by weighing wet resin with a mass of 2 g, followed by drying it in an oven (Venticell Eco Line, MMM Medcenter, Planegg, Germany) at 60 °C overnight. The determination was performed in duplicate.

#### 2.2.2. Adsorption Studies

The concentration of the colored compounds in the arabinoxylan extract solution was determined using the peak absorbance (λ = 283 nm) measured with a spectrophotometer (Evolution 201, Thermo Scientific, Waltham, MA, USA). A calibration curve was obtained by preparing a stock solution with a concentration of 1 g L^−1^ of freeze-dried material, followed by dilution to different concentrations and measurement of the respective absorbance at λ = 283 nm.

The adsorption capacity (*q*, mg g^−1^) was calculated using Equation (1):

(1) $$q=\frac{V\left(C_{0}-C_{e}\right)}{W}$$

where *V* (L) is the volume of the solution, *C*_0_ and *C_e_* are the initial and equilibrium concentrations (mg L^−1^), respectively, and *W* (g) is the resin dry weight.

The adsorption efficiency, E (%), was determined using Equation (2):

(2) $$E\;\left(\%\right)=\frac{C_{0}-C_{e}}{C_{0}}\cdot 100$$

- Effect of pH

Since lowering the pH of the arabinoxylan extract would cause the precipitation of unknown compounds, adsorption studies on the decolorization of the arabinoxylan solution were carried out without changing the pH of the original extract (pH = 7).

- Effect of Resin Dosage

To assess the influence of the resin dosage during the adsorption process, different resin dosages ranging between 0.005 and 0.2 g_dry resin_ mL_solution_^−1^ were added to a 2.5 g L^−1^ arabinoxylan solution at pH 7. The solution was left for 24 h at 25 °C with orbital stirring at 500 rpm (ISLD04HDG, Ohaus, Almada, Portugal). All the experiments were performed in triplicate, and the data was expressed as the mean ± the standard deviation.

- Resin Regeneration

The regeneration of the resin was assessed by performing adsorption and desorption cycles with the arabinoxylan extract. Regeneration was performed by mixing the wet resin with 10 mL of pure ethanol (0.2 g/10 mL) for 24 h and rinsing it afterwards with distilled water [11,19]. The adsorption efficiency was calculated after each adsorption/regeneration cycle, and four consecutive cycles were performed. All the experiments were performed in triplicate, and the data was expressed as the mean ± the standard deviation.

#### 2.2.3. Adsorption Isotherms

The experiments determining the adsorption isotherms of the arabinoxylan solutions consisted of varying the initial arabinoxylan concentration of the solution while maintaining the pH and resin dosage. To obtain equilibrium adsorption isotherms for the removal of the colored compounds from the arabinoxylan solutions (at concentrations of the colored compounds between 278 and 19,334 mg L^−1^), a constant dosage of 0.1 g_dry resin_ mL_solution_^−1^ was used with orbital stirring for 24 h at 25 °C, without changing the solution pH. All the experiments were performed in triplicate, and the data was expressed as the mean ± the standard deviation. The experimental data obtained for the adsorption of the colored compounds from the arabinoxylan extract solutions onto the resin MN102 were modeled using the Langmuir and Freundlich equations to study the interactions between the adsorbate and adsorbent [20].

The Langmuir model is expressed by Equation (3):

(3) $$q=\frac{q_{m}K_{L}C_{e}}{1+{K_{L}C}_{e}}$$

where *K_L_* (L mg^−^^1^) is the Langmuir adsorption equilibrium constant.

The Freundlich model is shown in Equation (4):

(4) $$q=K_{F}C_{e}^{\frac{1}{n}}$$

where *K_F_* (L mg^−^^1^) is the Freundlich constant and *n* is the heterogeneity factor.

### 2.3. Fixed-Bed Column Dynamic Adsorption Experiments

The experiments determining the arabinoxylan extract’s dynamic adsorption were performed in a glass column filled with 3 g of glass beads (2 mm diameter) used as support for the resin and a constant mass of 15 g of wet resin (5.75 g of dry resin). The column was packed with a bed height of 11 cm (Table 1), and the bed volume (BV) was calculated by multiplying the bed height by the cross-sectional area. A 1 g L^−1^ solution was introduced from the bottom, with the desired flow rates regulated using a peristaltic pump (Miniplus 3, Gilson, Lisbon, Portugal). Samples were collected from the top of the column at predetermined intervals to analyze the remaining colored compounds’ concentration. The breakthrough (t_b_), central (t_c_) and exhaustion (t_e_) times were defined as the times at which the normalized concentrations (C_out_/C_feed_) were 0.05, 0.50 and 0.95, respectively.

The influence of the contact time between the MN102 resin and the arabinoxylan extract solution was studied by using different flow rates (2.5, 5 and 10 mL min^−1^), maintaining the inlet concentration at 1 g L^−1^.

- Breakthrough curve modeling

Several mathematical models were used for the simulation of the dynamic behavior of the fixed-bed adsorption of the colored compounds. In this study, a general logistic function, the Yan model and the log-Gompertz model were fitted to the experimental data to predict the concentration profiles. Data fitting was carried out using Origin Pro 2022 software (OriginLab, Northampton, MA, USA). The adjusted coefficients of determination (R^2^) and reduced chi-squared values (χ^2^) were obtained using the software and employed in the evaluation of the validity of the breakthrough models.

- General logistic model

The Bohart–Adams, Thomas and Yoon–Nelson models have been extensively applied in dynamic adsorption behavior studies. However, research in the field has demonstrated that the aforementioned breakthrough models are, in fact, mathematically equivalent and can be reduced to a general logistic function [21]. This congruence simplifies the parameter determination process to a single nonlinear equation (Equation (5)).

(5) $$\frac{C}{C_{0}}=\frac{1}{1+e^{a-bt}}$$

where *a* and *b* represent different parameters of the breakthrough models. This general model has been validated against experimental breakthrough data by several authors [22]. The parameters of the simplified Bohart–Adams, Thomas and Yoon–Nelson models can be derived from the equations outlined in Table 2.

*k_BA_* (L mg^−1^ min^−1^), *k_T_* (L mg^−1^ min^−1^) and *k_YN_* (min^−1^) are rate coefficients, *N*_0_ is the saturation concentration of the colored compounds adsorbed in the packed bed (mg L^−1^), Z is the fixed bed’s length (cm), u is the superficial velocity (cm min^−1^), *q_o_* is the colored compounds’ adsorption capacity (mg g^−1^), m is the adsorbent mass (g), Q is the volumetric flow rate (L min^−1^) and τ is the time (min) required to achieve a 50% breakthrough.

- Yan model

The Yan model, proposed by Yan et al., 2001 [23], is also commonly described as the modified dose–response (MDR) model (Equation (6)). This model addresses problems associated with the Thomas model, which assumes a symmetric breakthrough curve and inaccurately describes the initial data [24,25].

(6) $$\frac{C}{C_{0}}=1-\frac{1}{1+{\left(\frac{V}{\beta }\right)}^{\alpha_{Y}}}$$

where *α* is the Yan constant and *β* is the volume at *C/C*_0_ = 0.5.

Applying the Thomas model and assuming that *C/C*_0_ = 0.5, the Yan model can be rewritten as follows:

(7) $$\frac{C}{C_{0}}=\;1-\frac{1}{1+{\left(\frac{C_{0}Q}{q_{0Y}m}t\right)}^{\alpha_{Y}}}$$

where *α_Y_* is the dimensionless Yan constant and *q*_0*Y*_ (mg/g) is the adsorption capacity.

- log-Gompertz model

The log-Gompertz model (Equation (8)) is a recent dynamic adsorption model that is useful for describing asymmetrical breakthrough curves, taking into account a floating inflection point [26,27].

(8) $$\frac{C}{C_{0}}=e^{{-e}^{k_{G1}-k_{G2}\ln t}}$$

where *k_G_*_1_ and *k_G_*_2_ (min^−1^) are the log-Gompertz constants and t is the time (min). Although the adsorption mechanisms cannot be inferred from this model, scaled-up process studies are still possible if the impact of the operational conditions on the model’s parameters is considered. For instance, the parameters increase with an increasing bed height but decrease with an increasing flow rate [28].

### 2.4. Film Formulation and Characterization

After the adsorption process, the solution was separated from the resin by vacuum filtration and freeze-dried to recover the remaining arabinoxylan.

#### 2.4.1. Arabinoxylan Film Preparation

Films were prepared by dissolving arabinoxylan (without contact with the resin and after contact with the resin) in deionized water at a concentration of 2% (*w*/*v*), and glycerol (30% (*w*/*w*_Ax basis_)) was used as a plasticizer. A total of 10 mL of the solution was poured into 50 mm plastic Petri dishes and left in the oven at 40 °C to dry.

#### 2.4.2. Film Characterization

- Color measurement

The characterization of the film color was performed using the CIELAB color system. This system quantifies the color using three parameters: L*, representing the lightness (ranging from 0 for black to 100 for white), and the chromaticity coordinates a* (positive for red, negative for green) and b* (positive for yellow, negative for blue). The values for these parameters were acquired using a digital colorimeter (Chroma meter CR-400, Konica Minolta, Tokyo, Japan), with measurements taken at 3–6 distinct points on each film.

The hue (*h°*), representing the angle on the chromaticity axis, was calculated using Equations (9) and (10).

(9) $$h{}^{\circ}=\arctan\frac{b^{*}}{a^{*}}\times \frac{180}{\pi }for\;a^{*}\;and\;b^{*}>0$$

(10) $$h{}^{\circ}=\left(\arctan\frac{b^{*}}{a^{*}}\times \frac{180}{\pi }\right)+180\;for\;a^{*}<0$$

The chroma (C*) or color saturation was calculated using Equation (11).

(11) $${C^{*}=\left({\left(a^{*}\right)}^{2}+{\left(b^{*}\right)}^{2}\right)}^{\frac{1}{2}}$$

The color difference (ΔE*_ab_), which considered the changes in L*, a* and b* between, for example, two samples, was calculated using Equation (12).

(12) $${∆E}_{ab}^{*}={\left({\left({∆L}^{*}\right)}^{2}+{\left({∆a}^{*}\right)}^{2}+{\left({∆b}^{*}\right)}^{2}\right)}^{\frac{1}{2}}$$

- Solubility in water

The solubility of the films was assessed using the following procedure, as described by Weng et al., 2021 [6]. Samples (10 × 10 mm) were prepared in triplicate. These were first dried at 50 °C for 24 h in an oven, and their initial dry mass (m_1_, g) was obtained. Each dried sample was then immersed in 5 mL of deionized water and orbitally agitated at 150 rpm for 24 h at 25 °C (room temperature). After the immersion period, the residual material was collected by centrifugation at 6000 rpm for 10 min, and the liquid phase was discarded. This collected solid residue was then dried again at 50 °C for 24 h, and its final dry mass (m_2_, g) was measured. The solubility (S, %) was calculated using Equation (13):

(13) $$S\;\left(\%\right)=\;\frac{m_{1}-m_{2}}{m_{1}}\;\times 100$$

- Mechanical Properties—Perforation

Before performing the perforation, the test films were stabilized in a desiccator with a relative humidity of 50% for a week. The tests were performed in a TA-XT plus texturometer (Stable Micro Systems, Surrey, UK) loaded with a 30 kg cell; a calibration weight of 5 kg was used, the trigger force was 0.049 N, the target mode was the distance until perforation, the pre-test speed was 1 mm s^−1^ and the post-test speed was 10 mm s^−1^, as described by Weng et al., 2021 [6]. The thickness of the films was measured with a digital micrometer (Digimatic Micrometer, Mitutoyo, Kanagawa, Japan). The samples were fixed above a support with a 1 cm diameter central aperture. A 2 mm diameter cylindrical probe then perforated the samples at a constant velocity of 1 mm s^−1^. The perforation stress (σ, MPa) was calculated by dividing the maximum perforation force (F, N) by the probe’s circular cross-sectional area (A, m^2^). The film deformation upon perforation (ε, %) was calculated using a geometric approach. The initial length was defined as the radius of the support hole. The distance the probe traveled until perforation was considered one side of a right-angled triangle, with the initial length as the adjacent side. The resulting hypotenuse represented the stretched length of the film. The deformation was then calculated as the ratio of the change in length (stretched length minus the initial length) to the initial length, expressed as a percentage. All the tests were conducted in 4–8 replicates.

- Statistical Analysis

Origin Pro 2019b (OriginLab, Northampton, MA, USA) was used to perform an analysis of variance (ANOVA), and the Tukey test (*p* value of 0.05) was used in order to detect differences among the mean values of the films’ properties.

## 3. Results and Discussion

### 3.1. Arabinoxylan Extract Studies

Preliminary studies of resin adsorption of the arabinoxylan extract at a neutral pH and pH 3.5 showed the precipitation of unknown compounds when the pH was lowered. This phenomenon hindered the adsorption process as well as the compound quantification; therefore, the arabinoxylan extract adsorption studies were carried out without altering the pH.

#### 3.1.1. Effect of Resin Dosage

**Figure 1:**
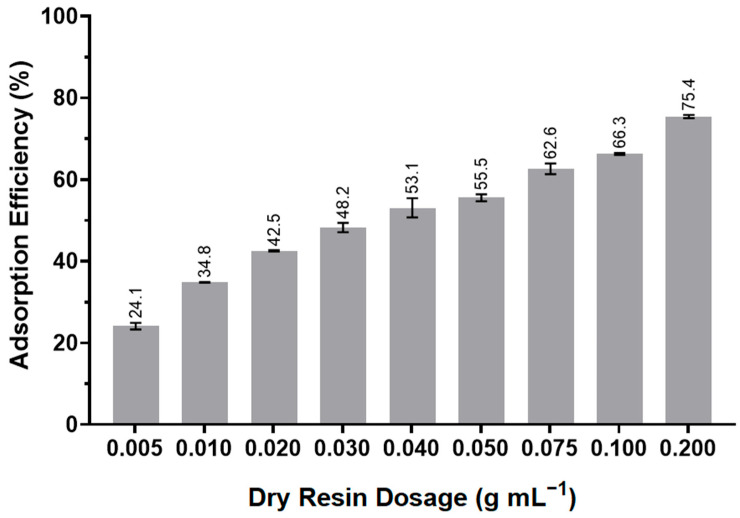
Adsorption efficiency (E%) as a function of the dry resin dosage (g_dry resin_ mL_solution_^−1^) in an arabinoxylan extract solution with a concentration of 2.5 g L^−1^. The error bars indicate the standard deviation of the means, *n* = 3.

A solution with a concentration of 2.5 g L^−1^ of the arabinoxylan extract was prepared, and different dry resin dosages were added to evaluate the effect of the resin dosage. The adsorption efficiency after 24 h with orbital stirring is presented as a function of the dry resin dosage in Figure 1.

Increasing the dry resin dosage led to an increase in the adsorption efficiency. Since the extract solution was more viscous than a simple aqueous solution, vigorous orbital stirring was needed. When the dry resin dosage was doubled, there was an increase of around 10% in the adsorption efficiency. For example, when the concentration doubled from 0.005 g_dry resin_ mL_solution_^−1^ to 0.01 g_dry resin_ mL_solution_^−1^, the efficiency also increased from 24.1% to 34.8%. The same occurred when the resin dosage was increased from 0.05 g_dry resin_ mL_solution_^−1^ to 0.1 g_dry resin_ mL_solution_^−1^, as the efficiency improved from 55.5% to 66.3%. At the highest tested resin dosage (0.2 g_dry resin_ mL_solution_^−1^), the solution could not be stirred properly due to the excess amount of resin present. Moreover, the viscosity of the solution impeded the proper stirring of the mixture, which may have negatively impacted the adsorption efficiency of the resin. The amount of resin necessary to reach substantial efficiency in the adsorption of the arabinoxylan extract was significant; however, it is expected that the resin could be recovered and recycled in new adsorption processes. Therefore, the concentration we chose to proceed with for the remaining experiments was 0.1 g_dry resin_ mL_solution_^−1^, achieving an adsorption efficiency of 66.3%. Dong et al. (2021) [13] reported 63% decolorization of crude grape pomace polysaccharides through adsorption with HPD-300 resin at a higher dosage of 0.2 g mL^−1^.

**Figure 2:**
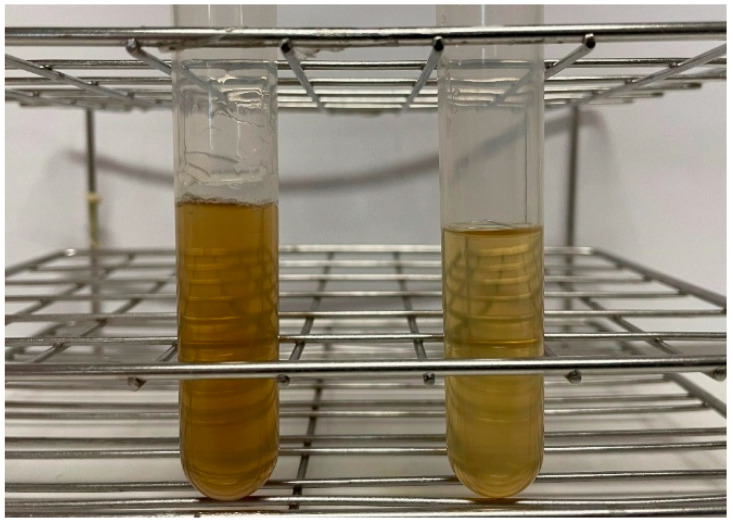
Arabinoxylan solution (2.5 g L^−1^) before (**left**) and after (**right**) 24 h of resin contact, with an adsorption efficiency of 66.3%.

The difference in the color of the tested arabinoxylan solution before and after the resin contact is shown in Figure 2. The contact of the arabinoxylan extract with the resin MN102 resulted in a high improvement in the color of the solution, which shifted to a lighter color, meaning that the adsorption of the colored compounds was successful. To achieve a higher degree of decolorization, consecutive adsorptions would be necessary.

#### 3.1.2. Resin Regeneration

**Figure 3:**
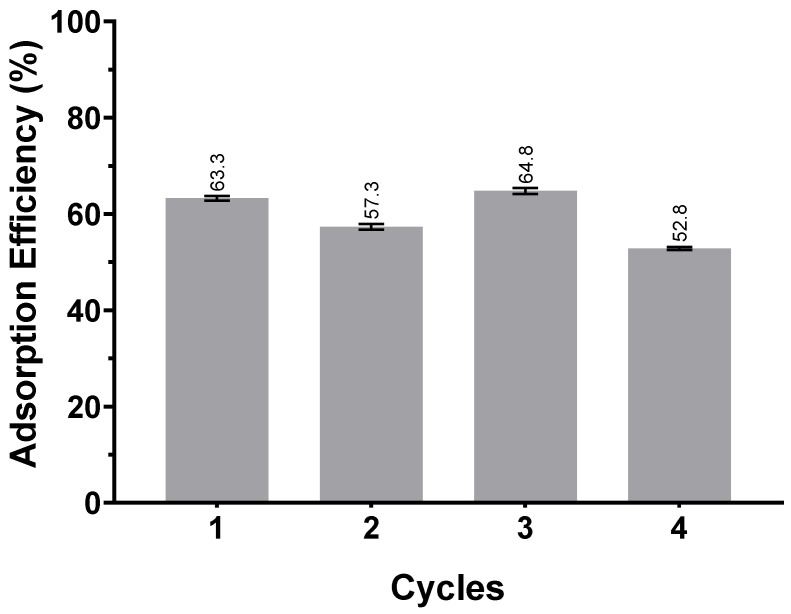
Adsorption efficiency (E%) after each adsorption/desorption cycle. The error bars indicate the standard deviation of the means, *n* = 3.

Adsorbent regeneration is an essential aspect to consider when designing an adsorption system. A great advantage is the adsorbent’s use in another cycle of adsorption. The recovery of the resin MN102 was studied by performing an adsorption experiment with the arabinoxylan solution, followed by desorption with pure ethanol. This cycle was repeated four times, and the adsorption efficiency was calculated after each adsorption cycle. A summary of the results obtained after the adsorption/desorption cycles is shown in Figure 3.

In the first cycle of adsorption, the resin came into contact with a solution of the arabinoxylan extract, and the adsorption efficiency was 63.3%. After the desorption process, the resin came into contact with a new solution of the arabinoxylan extract, and the efficiency slightly decreased to 57.3%. By the fourth cycle, the efficiency was 52.9%, meaning that the resin did not see a sharp decrease in its efficiency after four cycles of adsorption and desorption.

As mentioned before, although the amount of resin used for adsorption was significant (0.1 g_dry resin_ mL_solution_^−1^), the efficiency of the resin after the four cycles of adsorption followed by desorption only decreased slightly. Therefore, it is possible to reuse the same resin several times in new adsorption processes, which will compensate for the amount used in the process.

#### 3.1.3. Adsorption Isotherm Modeling

**Figure 4:**
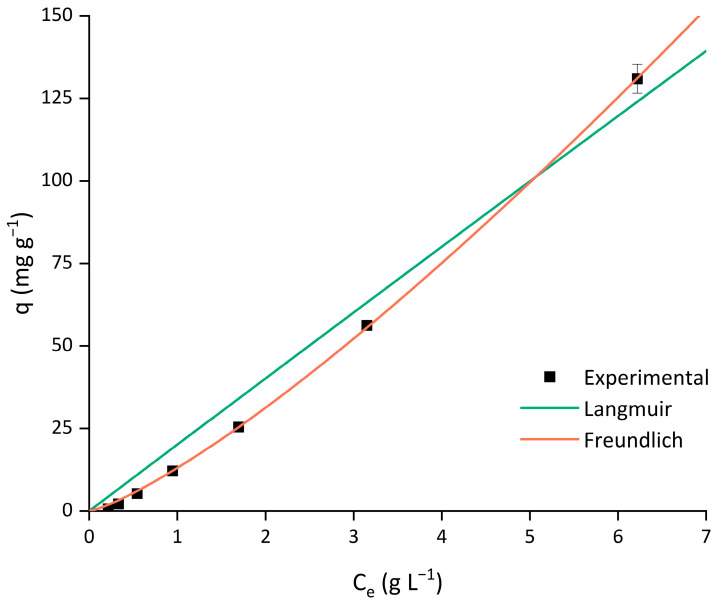
Adsorption isotherms of arabinoxylan extract solution with resin dosage of 0.1 g_dry resin_ mL_solution_^−1^. Error bars indicate standard deviation of means, *n* = 3.

Langmuir and Freundlich isotherm models were fitted to the experimental data obtained from the adsorption of the arabinoxylan extract. The data is presented in Figure 4.

The Langmuir model describes monolayer adsorption, assuming that only one molecule occupies each adsorption site; therefore, the adsorption is proportional to the number of sites already occupied. In contrast, the Freundlich model is based on multilayer adsorption, where adsorption occurs on heterogeneous surfaces, meaning that the binding sites and the adsorbates do not have homogeneous affinity [29].

It was expected that at higher concentrations, the adsorption capacity would reach a plateau in the Langmuir model and display a curve in the Freundlich model. As observed in Figure 4, the adsorption capacity increased almost linearly with the increase in the concentration, with an experimental maximum adsorption capacity value of 130.93 mg g^−1^. In this case, due to the viscosity of the arabinoxylan solution, it was not possible to increase the concentration of the solution further since that would prevent a uniform distribution of the resin while stirring it. The calculated parameters for the Langmuir and Freundlich isotherm models are represented in Table 3. When comparing the correlation coefficient values (R^2^), both models seemed to, to some degree, accurately represent the experimental data. However, the Freundlich model had a higher R^2^ value (R^2^ > 0.99). Additionally, the χ^2^ value (used to evaluate the goodness of fit, which considers the difference in the experimental data points and the values predicted by the model, meaning that a higher value indicates a larger deviation between the two) was 1.307 for the Freundlich model, while for the Langmuir model it was higher (χ^2^ = 14.73). Furthermore, observing the graphical representation, this model seemed to fit the experimental data better. To more accurately determine the correlation and applicability of the models, a study examining higher concentrations would be necessary.

#### 3.1.4. Dynamic Adsorption Experiments

In this part of the research, the effect of the flow rate on the adsorption of the colored compounds using MN102 resin was investigated at flow rates of 2.5, 5 and 10 mL min^−1^. The other operating parameters were kept constant; namely the columns were packed with 15 g of wet resin (5.75 g of dry resin) and the arabinoxylan solution (1 g L^−1^) was fed from the bottom to the top of the column.

**Figure 5:**
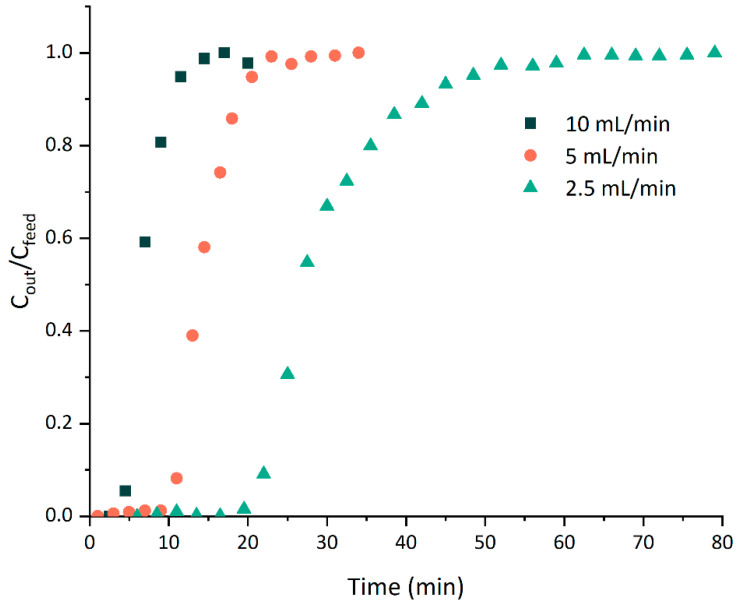
Breakthrough curves for fixed-bed adsorption of arabinoxylan extract colored compounds. The reported values are from a single experiment (*n* = 1).

Figure 5 shows the breakthrough curves obtained at different flow rates, expressed as the normalized concentration, the ratio between the outlet concentration and initial concentration (*C_out_/C_feed_*), as a function of the time (*t*). The obtained experimental breakthrough and exhaustion times (where *C_out_/C_feed_* was 0.05 and 0.95, respectively) are displayed in Table 4. The dynamic adsorption of the arabinoxylan solution followed a typical S-shaped breakthrough curve. The results show that the inlet flow rate had a significant effect on the breakthrough behavior. From Figure 5, it can be observed that increasing the flow rate led to steeper breakthrough curves, with lower breakthrough and exhaustion times (t_b_ and t_e_, respectively). With higher flow rates, the residence time in the fixed bed decreased and, consequently, the contact time between the resin and the feed solution was shorter. The breakthrough times obtained were 20.61, 10.53 and 4.45 min with corresponding BVs (1 BV = 54 mL) of 1.0, 1.0 and 0.8 for flow rates of 2.5, 5 and 10 mL min^−1^, respectively. Specifically, at 10 mL min^−1^, the resin was saturated earlier, resulting in a lower breakthrough capacity. So, as the flow rate increased from 2.5 to 10 mL min^−1^, the breakthrough time decreased to a quarter of its value. Similarly, the observed exhaustion times were significantly reduced with an increasing flow rate. For flow rates of 2.5, 5 and 10 mL min^−1^, the exhaustion times were 46.91, 21.64 and 12.24 min with a corresponding BV of 2.2, 2.0 and 2.3. Once again, when the flow rate rose from 2.5 to 10 mL min^−1^, the exhaustion time was reduced to a quarter of its value, meaning that a smaller contact time was required for the saturation of the resin.

#### 3.1.5. Breakthrough Curve Modeling

**Figure 6:**
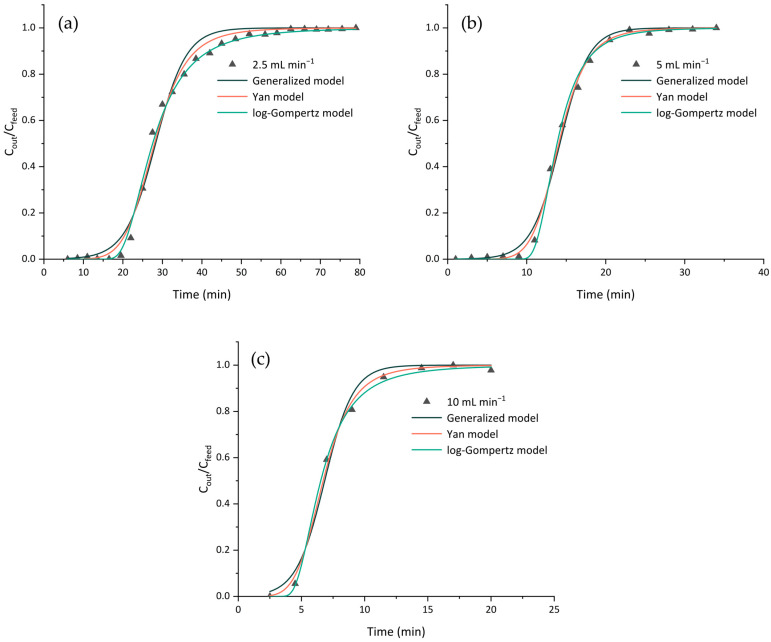
Experimental (symbols) and predicted (full lines) breakthrough curves obtained using the Yan, log-Gompertz and generalized logistic models at flow rates of (**a**) 2.5, (**b**) 5 and (**c**) 10 mL min^−1^. The reported values are from a single experiment (*n* = 1).

The dynamic adsorption behavior was fitted with the Yan model, log-Gompertz model and generalized logistic model, which, as mentioned previously, simplified the parameter determination of the Bohart–Adams, Thomas and Yoon–Nelson models. Figure 6 shows the breakthrough curves at different inlet flow rates and the applied breakthrough models. Table 5 and Table 6 summarize the parameters obtained for the dynamic adsorption models.

The parameters of the Bohart–Adams, Thomas and Yoon–Nelson models were calculated using Equation (5) ($\frac{C}{C_{0}}=\frac{1}{1+e^{a-bt}}$) and the parameters in Table 2, and their results are presented in Table 5. The three models share the same R^2^ and χ^2,^ owing to their mathematical equivalence to the generalized model. Therefore, from a mathematical perspective, the models cannot be compared in terms of their goodness of fit [30]. The experimental data was fitted with the general logistic model with a high R^2^ (0.988, 0.995 and 0.988) for the dynamic adsorption of the arabinoxylan extract at 2.5, 5 and 10 mL min^−1^. The kinetic constants, k_BA_ and k_T_, showed an upward trend with an increase in the flow rate. Moreover, the saturation concentration (N_0_) and adsorption capacity (q_0_) parameters were somewhat constant throughout the experiments, even when different flow rates were applied to the fixed-bed column. Finally, the time required to achieve 50% adsorbate breakthrough (τ), obtained from the Yoon–Nelson model (28.3 ± 0.4, 14.2 ± 0.9 and 6.8 ± 0.9 min for the flow rates of 2.5, 5 and 10 mL min^−1^, respectively), closely aligned with the experimental values (t_c_, Table 4; 27.5, 13.9 and 6.6 min for the flow rates of 2.5, 5 and 10 mL min^−1^, respectively).

Table 6 shows the parameters of the remaining dynamic adsorption models, the Yan and log-Gompertz models. In this case, the adsorption capacity (q_0_) obtained from the Yan model closely resembled the respective parameter when the Thomas model was applied (Table 5). This reinforces the notion that all the models were highly suitable for the fitting of the experimental data. Overall, all the models provided a good fit to the experimental data, with high coefficients of determination (R^2^) and low χ^2^ values. Nevertheless, the Yan and log-Gompertz models provided the most suitable fit to the experimental data, with R^2^ > 0.994 for all the breakthrough curves. Additionally, the near-zero χ^2^ values indicate that there were virtually no differences between the experimental and calculated variables. The better fit to the experimental breakthrough data with these models may be because, unlike the general logistic model (which includes the Bohart–Adams, Thomas and Yoon–Nelson models), the Yan and log-Gompertz models accurately describe asymmetric breakthrough curves, which were the kind obtained in this study. Similar findings were also reported by Oliveira et al. [28], where the authors stated that both the Yan and log-Gompertz models predicted the behavior of tetracycline adsorption in a granular activated carbon fixed-bed column.

#### 3.1.6. Scaled-Up Process

The scaled-up design of this adsorption system was strategically based on maintaining specific operational conditions to ensure comparable performance characteristics and preserve the fundamental dynamics of the adsorption process across different scales. For this demonstration, the system geometry, polymer dosage and feed solution concentration (1 g L^−1^) were fixed. A key principle in this scaling up approach is to maintain a constant solute velocity (u_s_, cm min^−1^), which is possible to achieve by using the same resin with the same characteristics, like the porosity, particle size and shape; by keeping this parameter constant, we could calculate the new central time (min) for a new bed height (L, cm) using Equation (14) below.

(14) $$t_{c}=\frac{L}{u_{s}}$$

Table 7 shows examples of the calculated parameters for different scale-up factors for a flow rate of 10 mL min^−1^. For a scale-up factor of 5, the new bed height would be 55 cm. Accordingly, the new central time would be 33 min. By maintaining these parameters, the new scaled-up process could achieve the same efficiency as that of the original experiment.

### 3.2. Arabinoxylan Film and Characterization

#### 3.2.1. Arabinoxylan Films

**Figure 7:**
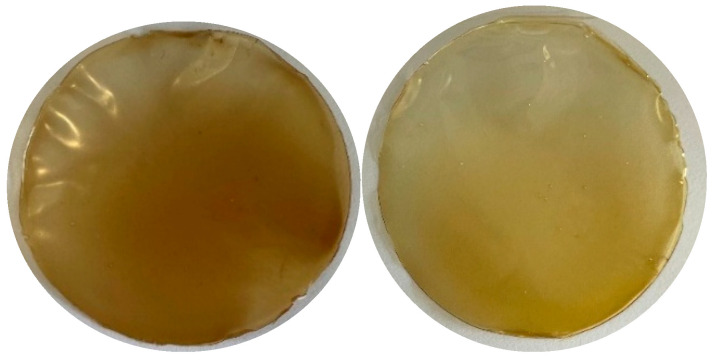
Formulated arabinoxylan film without resin contact (Ax, **left**) and after resin contact (AxR, **right**).

The films were prepared as described previously in Section 2.4.1, with 10 mL of the solution poured into 50 mm plastic Petri dishes. The resulting films are presented in Figure 7 below.

Both films had a smooth surface and were flexible; the arabinoxylan film without resin contact had a darker tone, while the film obtained after resin contact was lighter.

#### 3.2.2. Color Measurement

The obtained parameters regarding the color of the films are presented in Table 8 below.

The lightness values indicate that the AxR films (81.07 ± 0.61) were lighter (value closer to 100) when compared to the Ax film (68.32 ± 2.15). According to the chromaticity coordinates as well as the obtained hue values, both films fell in the range of yellow, with the AxR film being closer to a pure yellow color while the Ax film fell more in the range of an orange-yellow color. Additionally, the color saturation also slightly decreased from (34.03 ± 1.52) to (27.40 ± 1.62) during the adsorption process. The color difference (ΔE*_ab_) was calculated to quantify the changes between the films. The standard interpretation suggests that ΔE*_ab_ values from 0 to 1 are not noticeable to the human eye. Values exceeding 5 indicate that an observer perceives two different colors, and the greater this difference, the more opposite the colors are considered to be [31]. Given that the ΔE*_ab_ obtained for our samples was 15.39 ± 2.08, it is evident that a significant color difference existed between the films. Overall, this indicates that the decolorization process was somewhat successful, as observed previously.

#### 3.2.3. Solubility in Water

The solubility in water of the films was evaluated, and the obtained results are presented in Table 9.

Based on the results, the water solubility of the AxR film remained unaffected by the adsorption process. The Ax film showed a solubility of 96 ± 2%, while the AxR film had a solubility of 91 ± 5%. This suggests that the removal of the colored compounds during the adsorption process did not substantially impact the films’ interaction with water, leading to their almost complete dissolution. Bangar et al. (2021) prepared flaxseed-meal-based edible films with pectin, which showed high solubility (>98%), confirming their solubility in the mouth and possible application as edible films [32].

#### 3.2.4. Mechanical Properties

The obtained calculated mechanical parameters are summarized in Table 10.

As observed in Table 10, the thickness of the films did not significantly change after resin contact; additionally the tension of perforation for both films was very similar, 2.71 ± 1.23 and 2.55 ± 0.85 MPa for the Ax and AxR films, respectively. This indicated that the contact with the resin did not change the mechanical properties in terms of tension of perforation or the thickness of the film after being decolorized. On the other hand, the deformation upon perforation increased by around 1.6 times. This could be explained by the presence of colored compounds in the arabinoxylan matrix, which could have restricted the deformation of the Ax film until after the adsorbed part of these compounds was removed, allowing the arabinoxylan chain in the AxR film to move more freely.

## 4. Conclusions

In the present study, the ability of a macroporous resin (MN102) to adsorb an arabinoxylan extract solution was successfully tested. The adsorption was efficient at a neutral pH and resin dosage of 0.1 g_dry resin_ mL_solution_^−1^, which resulted in decolorization and a lighter colored solution. In addition, the recovery of the resin for use in a new adsorption process is possible, and after four adsorption/desorption cycles, the adsorption efficiency only slightly decreased from 63.3% to 52.9%. The arabinoxylan extract adsorption data was fitted by the Freundlich isotherm model. The dynamic adsorption behavior of the arabinoxylan extract was analyzed using several mathematical models. The results suggest that the Yan and log-Gompertz models, commonly applied to asymmetric breakthrough curves, offer the best fit for the adsorption of colored compounds from an arabinoxylan extract. A fourfold increase in the inlet flow rate (from 2.5 to 10 mL min^−1^) led to a decrease in the breakthrough and exhaust times to a fourth of their value. Overall, this study provides valuable insights into the use of resin MN102 for the removal of colored compounds. Additionally, it was observed that the adsorbed solution was able to retain its film-forming properties, producing stand-alone films which retained some of their main characteristics, namely their water solubility and their mechanical properties (perforation), with a slight increase in their deformation. Moreover, the color of the films shifted to a lighter yellow color as expected, which is more appealing for food packaging applications.

## Figures and Tables

**Table 1:** Parameters of the fixed-bed column used in the dynamic adsorption experiments.

Internal diameter, D_c_ (cm)	2.50
Total column length, L_tot_ (cm)	20.00
Wet resin weight (g)	15.00
Dry resin mass (g)	5.75
Bed height, L_c_ (cm)	11.00
Bed volume, BV (mL)	54.00
L_c_/D_c_	4.40
Cross-sectional area, A_c_ (cm^2^)	4.91

**Table 2:** Summary of the relationship between the Bohart–Adams, Thomas and Yoon–Nelson models, with parameters *a* and *b* of the logistic function.

Model	*a*	*b*
Bohart–Adams	$\frac{k_{BA}N_{0}Z}{u}$	$k_{BA}C_{0}$
Thomas	$\frac{k_{T}q_{0}M}{Q}$	$k_{T}C_{0}$
Yoon–Nelson	$k_{YN}\tau$	$k_{YN}$

**Table 3:** Adsorption isotherm parameters of arabinoxylan extract solution with resin dosage of 0.1 g_dry resin_ mL_solution_^−1^.

Sample	Langmuir				Freundlich			
	q_max_ (mg g^−1^)	K_L_ (L mg^−1^)	R^2^	χ^2^	n	K_F_ (L mg^−1^)	R^2^	χ^2^
Arabinoxylan Extract	1.093 × 10^4^	1.845 × 10^−6^	0.9789	14.73	0.7924	2.138 × 10^−3^	0.9997	1.307

**Table 4:** Summary of experimental breakthrough, central and exhaustion times for different flow rates.

Flow Rate (mL min^−1^)	2.5	5	10
t_b_ (min)	20.6	10.5	4.5
t_c_ (min)	27.5	13.9	6.6
t_e_ (min)	46.9	21.6	12.2

**Table 5:** Parameters of the general logistic model under different flow rates.

		Flow Rate (mL min^−1^)		
Model	Parameter	2.5	5	10
General logistic	a	7.25 ± 0.70	7.78 ± 0.62	6.03 ± 0.98
	b (min^−1^)	0.257 ± 0.025	0.548 ± 0.043	0.881 ± 0.139
	χ^2^	0.002	0.001	0.002
	R^2^	0.988	0.995	0.988
Bohart–Adams	k_BA_ × 10^3^ (L mg^−1^ min^−1^)	0.224 ± 0.022	0.435 ± 0.034	0.725 ± 0.114
	N_0_ (mg L^−1^)	1500.2 ± 145.4	1656.5 ± 131.3	1539.0 ± 250.7
Thomas	k_T_ × 10^3^ (L mg^−1^ min^−1^)	0.224 ± 0.022	0.435 ± 0.034	0.725 ± 0.114
	q_0_ (mg g^−1^)	14.1 ± 1.4	15.6 ± 1.2	14.5 ± 2.4
Yoon–Nelson	k_YN_ (min^−1^)	0.257 ± 0.025	0.548 ± 0.043	0.881 ± 0.139
	τ (min)	28.3 ± 0.4	14.2 ± 0.9	6.8 ± 0.9

**Table 6:** Parameters of the dynamic adsorption models for asymmetric breakthrough curves under different flow rates.

		Flow Rate (mL min^−1^)		
Model	Parameter	2.5	5	10
Yan	q_0_ (mg g^−1^)	13.9 ± 0.1	15.4 ± 0.0	14.2 ± 0.2
	α_Y_	6.89 ± 0.42	7.88 ± 0.03	5.73 ± 0.49
	χ^2^	0.001	0.000	0.001
	R^2^	0.994	0.999	0.996
log-Gompertz	k_G1_	14.5 ± 1.2	16.1 ± 1.5	7.1 ± 0.4
	k_G2_ (min^−1^)	4.5 ± 0.4	6.3 ± 0.6	4.0 ± 0.2
	χ^2^	0.000	0.000	0.000
	R^2^	0.997	0.998	0.999

**Table 7:** Calculated scaled-up parameters for the dynamic adsorption of arabinoxylan at a flow rate of 10 mL min^−1^.

Scale-Up Factor	Bed Height (cm)	t_c_ (min)
1	11	6.6
5	55	33
10	110	66

**Table 8:** Values of the lightness (L*), chromaticity coordinates (a*, b*) and the calculated hue (*h°*) and chroma (C*) for the arabinoxylan film without resin contact (Ax) and the arabinoxylan film after resin contact (AxR). Values in the same column followed by different superscript letters differed significantly (*p* < 0.05). The error bars indicate the standard deviation of the means, *n* = 3–6.

Film	L*	a*	b*	*h°*	C*	ΔE*_ab_
Ax	68.32 ± 2.15 ^a^	5.72 ± 1.08 ^a^	33.54 ± 1.36 ^a^	80.36 ± 1.44 ^a^	34.03 ± 1.52 ^a^	15.39 ± 2.08
AxR	81.07 ± 0.61 ^b^	−0.35 ± 0.20 ^b^	27.40 ± 1.62 ^b^	90.75 ± 0.46 ^b^	27.40 ± 1.62 ^b^	

**Table 9:** Solubility in water (%) of the arabinoxylan film without resin contact (Ax) and arabinoxylan film after resin contact (AxR). Values in the same column followed by different superscript letters differed significantly (*p* < 0.05). The error bars indicate the standard deviation of the means, *n* = 3.

Film	Solubility (%)
Ax	96 ± 2 ^a^
AxR	91 ± 5 ^a^

**Table 10:** Thickness (µm), tension of perforation (σ, MPa) and deformation upon perforation (ε, %) of the arabinoxylan film without resin contact (Ax) and arabinoxylan film after resin contact (AxR). Values in the same column followed by different superscript letters differed significantly (*p* < 0.05). The error bars indicate the standard deviation of the means, *n* = 4–8.

Film	Thickness (µm)	Tension of Perforation (σ, MPa)	Deformation Upon Perforation (ε, %)
Ax	81 ± 13 ^a^	2.71 ± 1.23 ^a^	32.0 ± 4.5 ^a^
AxR	69 ± 20 ^a^	2.55 ± 0.85 ^a^	52.4 ± 5.4 ^b^

## Data Availability

The data will be made available on request.
